# Fostering preservice teachers’ expectancies and values towards computational thinking

**DOI:** 10.3389/fpsyg.2022.987761

**Published:** 2022-09-28

**Authors:** Anke M. Weber, Morten Bastian, Veronika Barkela, Andreas Mühling, Miriam Leuchter

**Affiliations:** ^1^Computer-Based Assessment Research Group, Department of Behavioural and Cognitive Sciences, University of Luxembourg, Esch-sur-Alzette, Luxembourg; ^2^Computing Education Research Group, Department of Computer Science, University of Kiel, Kiel, Germany; ^3^Department of Educational Sciences, Institute for Children and Youth Education, University of Koblenz-Landau, Landau, Germany

**Keywords:** expectancy-value theory, computational thinking, preservice teacher education, primary school, values, emotional costs, expectancies of success

## Abstract

**Theory:**

Digital technologies have become an integral part of everyday life that children are exposed to. Therefore, it is important for children to acquire an understanding of these technologies early on by teaching them computational thinking (CT) as a part of STEM. However, primary school teachers are often reluctant to teach CT. Expectancy-value theory suggests that motivational components play an important role in teaching and learning. Thus, one hindrance to teachers’ willingness to teach CT might be their low expectancies of success and high emotional costs, e.g., anxiety towards CT. Thus, introducing preservice teachers to CT during their university years might be a promising way to support their expectancies and values, while simultaneously alleviating their emotional costs. Prior CT competences might contribute to these outcomes.

**Aims:**

We investigated whether a specifically designed seminar on CT affected preservice teachers’ expectancies and values towards programming.Method: A total of 311 German primary school and special education preservice teachers took part in the study. The primary school preservice teachers received a seminar on CT and programming with low-threshold programming tasks, while the special education teachers served as a baseline group. The seminar was specifically designed to enhance expectancies and values and decrease emotional costs, following implications of research on expectancy-value theory.

**Results:**

The preservice teachers who visited the seminar gained higher expectancies and values towards CT and programming compared to the baseline group. Moreover, their emotional costs decreased. CT was positively related to change in expectancies and values and negatively related to emotional costs.

**Discussion:**

Interventions with low-threshold programming tasks can support primary school preservice teachers in finding trust in their abilities and values towards CT. Moreover, their anxiety towards CT and programming can be alleviated. Thus, first steps in preparing preservice teachers to teach CT in their future classrooms can be taken in university.

## Introduction

Today’s society is becoming increasingly digitized which means that children come into contact with and use programmed devices such as tablets, smartphones, and computers at an early age. Therefore, it is important that even young children acquire an understanding of these technologies. More specifically, children should not only be able to use these technologies, but also have a basic understanding of how they work (Barr and Stephenson, 2011). Solving problems with the help of computers, e.g., by writing a program, is commonly referred to as computational thinking (CT). More specifically, CT encompasses cognitive processes that formulate problems and their solutions so that they can be processed by an information processing agent (e.g., a computer or a processor; Newell et al., 1959; Wing, 2006; Faber et al., 2017; Shute et al., 2017; Weber et al., 2021).

The teaching of CT and its implementation as a part of STEM is called for by different stakeholders (e.g., Wing, 2006; National Research Council, 2010; Ministerium für Bildung, Wissenschaft, Jugend und Kultur, 2018; OECD, 2018). While advancements to implement CT into secondary education have been made, teaching CT at primary school level has seen fewer efforts (Angeli et al., 2016). However, learning CT skills early might be beneficial as it might foster children’s problem-solving skills as a part of their 21st century skills (Barr and Stephenson, 2011; Jaipal-Jamani and Angeli, 2017; Yadav et al., 2017; Lamprou and Repenning, 2018). Moreover, it can support their metacognition and cognitive flexibility, which are goals of primary school education (National Research Council, 2010; Di Lieto et al., 2017; OECD, 2018). CT can be implemented into primary school classrooms by using simple block-based programming tasks or unplugged CT activities (Wing, 2006; Brackmann et al., 2017). For the most part programming tasks are used at this education level because children have fun programming robots or computers (Hsu et al., 2018) and solving problems through programming is a central part of CT (Román-González et al., 2017; Shute et al., 2017).

Teaching CT as early as in primary school is possible (Angeli et al., 2016; Dickes et al., 2016; Di Lieto et al., 2017). However, implementing CT poses challenges for primary school teachers. In order to teach CT, teachers require CT competences and knowledge of support measures in informatics problem solving that they often do not possess (see, TPACK; Angeli et al., 2016). Thus, it is desirable to implement CT education into preservice teacher education at the university level to target this challenge early on (Yadav et al., 2014; Butler and Leahy, 2021).

However, political, administrative, educational, and interindividual hindrances need to be overcome to integrate CT education into primary school (Angeli et al., 2016). This paper addresses some of these interindividual obstacles in teacher education because preservice teachers often are reluctant concerning the learning and teaching of CT (Gal-Ezer and Stephenson, 2010; Weber et al., 2021). Following expectancy-value theory (EVT; e.g., Eccles et al., 1983), one of the most influential motivational theories, reasons might include low expectancies of success as well as low values towards and fears of CT and programming. These fears might be heightened for women who make up a large percentage of primary school preservice teachers (OECD, 2021) due to stereotype threats (see Shapiro and Williams, 2012; Marsh et al., 2019).

Eccles (2009) claims that expectancies and values predict a person’s choices and decisions. As CT and programming grow ever more important, it is crucial that primary school teachers choose to teach these important topics to young learners and actively decide to implement them into their classrooms. Teachers might be more likely to teach this content if they perceive themselves as able, value CT, and are under the impression that it is important for children to learn basic CT competences. Therefore, one step to take in order to address interindividual obstacles that stand in the way of CT education might be to implement a preservice teacher education program focusing on CT. This program can encompass measures that foster preservice teachers’ expectancies of success as well as positive values regarding CT. Consequently, this study investigates preservice teachers’ expectancies and values regarding CT as a part of STEM.

### Expectancy-value theory

According to EVT (e.g., Eccles et al., 1983), both the expectancy of success and the value a person places on a task or a domain influence persistence and the pursuit of plans (Bong et al., 2012; Musu-Gillette et al., 2015). Therefore, preservice teachers’ expectancies of success and values might be important for their willingness to implement complex STEM topics such as CT into their classrooms (Rich et al., 2021). Considering the challenges CT poses for primary school teachers (Yadav et al., 2014)–e.g., overcoming anxieties, having to learn a programming language–teachers with higher expectancies and values regarding the teaching of CT might be more likely to implement it in their classrooms.

#### Expectancies of success

Expectancies of success are often operationalized in the form of competence beliefs, such as self-concept and self-efficacy (Marsh et al., 2019). For example, Eccles (2009) has stated that expectancy of success and self-concept are linked in a way that makes it hard to distinguish between the two constructs. Therefore, self-concept can be viewed as one representative of a person’s expectancy of success in a domain. Self-concept refers to broad self-beliefs about one’s competence and includes self-evaluations that often draw on past experiences (Marsh et al., 2012). For example, a person with a high self-concept in CT might view programming – a central part of CT – as one of their strengths. Teachers might differ in their self-concept regarding CT and therefore some teachers might view themselves as rather good at programming, while others might consider themselves as rather bad at programming. For example, Faherty et al. (2021) found that secondary school teachers’ self-concepts in programming ranged from very low to very high, when asked about their programming skills. Moreover, Bouck et al. (2021) state that special education preservice teachers report relatively low self-concepts in teaching CT to students.

Another component indicative of expectancy of success is self-efficacy as a specific belief about certain tasks within a domain (Trautwein et al., 2013; Wigfield et al., 2017). In contrast to self-concept, self-efficacy is often construed as a belief that is more descriptive in nature and refers to tasks that lie in the future (Marsh et al., 2012). For example, a person with a high self-efficacy in CT might determine that they will be able to solve specific programming tasks, such as programming loops or conditions. Teachers might differ in their self-efficacy regarding CT as well. Thus, some might be quite confident that they can solve specific programming tasks. Others might hold more reluctant beliefs about their ability to solve the same tasks. Studies by Boulden et al. (2021) and Rich et al. (2021) found that primary school preservice teachers held different self-efficacy beliefs towards programming and the teaching of CT that were not influenced by their demographic characteristics. Moreover, Zhao et al. (2020) discovered that teachers’ self-efficacy beliefs concerning the teaching of CT increased during a three-week Scratch online course.

#### Values

Following Eccles (2009), there are four different value components, intrinsic value, utility value, attainment value, and costs. Intrinsic value is conceptualized as intrinsic motivation, i.e., the enjoyment of a certain task. Teachers might differ in their enjoyment of programming. Jaipal-Jamani and Angeli (2017) found that primary school preservice teachers mostly reported that they enjoyed learning about programming in the context of robotics. Moreover, their enjoyment increased after taking part in a programming activity with educational robots.

Utility value refers to the usefulness of something to achieve short-or long-term goals that a person has (Wigfield and Cambria, 2010; Marsh et al., 2019). Teachers might differ in their perception of needing to learn the basics of CT and programming themselves, based on their own willingness to teach CT and programming to young learners. Jaipal-Jamani and Angeli (2017) investigated whether primary school preservice teachers would teach CT in their classrooms. They found that most preservice teachers disagreed to teaching CT. However, their values regarding the teaching changed towards a more positive attitude after participating in a robotics programming task.

Attainment value describes the personal value a person attributes to something (Eccles, 2009). Teachers might differ in whether they value programming or the development of CT competences as an important learning goal for children. In a qualitative study with special education preservice teachers, Bouck et al. (2021) report that most preservice teachers value the importance of teaching CT to their students. This might also be the case for primary school preservice teachers.

Last, costs imply the negative consequences a certain task might have, e.g., emotional costs such as anticipated anxiety. Teachers might differ in their anxiety towards programming and CT. In the context of programming, Nolan and Bergin (2016) found that even computer science students often are anxious about programming. This anxiety might be even higher for primary school teachers or preservice teachers (Weber et al., 2021), because they are mostly female. Studies found that women often tend to think they have low abilities in STEM (Shapiro and Williams, 2012; Marsh et al., 2019). Moreover, CT courses are often absent from teachers’ university education (Angeli et al., 2016), which might contribute to their anxiety towards the subject.

The studies cited above did not specifically investigate EVT and all its components. As both expectancy and value can influence persistence and the pursuit of plans (Bong et al., 2012; Musu-Gillette et al., 2015), it is worthwhile to investigate preservice primary school teachers’ expectancies of success and values towards programming as indicators for their later willingness to teach CT to young learners. Additionally, their EVT components might be changed during a specifically designed intervention (O'Mara et al., 2006; Dowker et al., 2016; Wigfield et al., 2017).

### Designing a university seminar to support expectancies and values

Wigfield and his colleagues (Guthrie et al., 2004; Wigfield and Guthrie, 2010; Wigfield et al., 2017) found that students’ expectancies of success and values towards different learning outcomes can be fostered with specifically designed interventions. Such interventions that target expectations and values, as is demanded by Nagengast et al. (2011), should include six characteristics. (1) Interventions should have content goals. These can help students structure their learning and provide them with a sense of purpose by making them aware why they are completing certain tasks or why they need to read a certain article (van de Pol et al., 2010; Belland et al., 2013). For example, the lecturer can explain why and how a task supports students’ programming competences. (2) Students should be able to make choices and have control over the tasks whenever possible. This can support their desire for autonomy and prevent them from experiencing frustrations (Wigfield and Tonks, 2004). Even if students need to complete all tasks for a seminar, they can be offered control over the framework conditions (van de Pol et al., 2010). For example, students can decide when to complete a task and which resources to use. (3) Hands-on activities might help students to realize the value of what they study. For example, completing low-threshold programming tasks might allow students to appreciate that CT can be implemented into their future classrooms even with young learners (Angeli et al., 2016; Sengupta et al., 2018; Weber et al., 2021). This might in turn enhance their values towards CT and programming as preservice teachers take great interest in example tasks that they can easily implement later in their career (Garet et al., 2001). (4) Interesting tasks should be used for instruction to enhance students’ intrinsic value to engage with the subject matter. For example, a programming task that can easily be implemented in a future classroom and consists of everyday activities or objects, e.g., programming a bicycle lamp, serves this purpose (Angeli et al., 2016; Reuter et al., 2021). Again, such tasks are interesting especially for preservice teachers (Garet et al., 2001). (5) Collaborations between students regarding the learning objective can increase students’ values towards learning through working with others (Wigfield et al., 2014). For example, students can program in pairs or groups. Moreover, a seminar lecturer can offer the students the possibility of exchange in an online forum (Kong et al., 2018). (6) The importance of the learning objective should be emphasized to foster students’ value beliefs. Students should also be encouraged to reflect about the importance themselves in order to value their autonomy (Wigfield et al., 2017). Thus, a lecturer can highlight the importance of learning CT for young children and therefore the need for preservice teachers to learn it as well (Yadav et al., 2014). The students can then reflect on this themselves.

### Required content knowledge about CT for the primary school classroom

To teach CT in future primary school classrooms, preservice teachers need TPACK (technological pedagogical content knowledge). TPACK encompasses a basic understanding of CT, at least rudimentary programming skills, and knowledge of pedagogical approaches to teach CT to young learners (Angeli et al., 2016). To acquire knowledge about CT and programming, block-based visual programming languages like Scratch or NEPO® are suited, because they lighten the burden of writing syntactical correct programs (Resnick et al., 2009).

CT is a set of thought processes in which problems and their solutions are formulated in a way that can be processed by an information processing agent (e.g., a computer; a processor; Newell et al., 1959; Wing, 2006; Faber et al., 2017; Shute et al., 2017; Weber et al., 2021). Thus, CT encompasses a set of cognitive processes, such as decomposing problems into subproblems, abstracting, sequencing algorithms, control flow, debugging and generalizing. These processes enable learners to solve problems in a specific way, i.e., by creating algorithms (White and Sivitanides, 2003; Wing, 2006; National Research Council, 2010; Angeli et al., 2016). To plan a solution, a given problem needs to be decomposed into subproblems to reduce complexity. Thus, the decomposition will facilitate the understanding and the solving of the problem (Newell et al., 1959), e.g., because solution strategies for the subproblems might already be known (Faber et al., 2017). Abstraction excludes irrelevant properties of a problem to expose the underlying algorithm and can make problem-solving activities more efficient, given that certain algorithms represent certain subproblems (Jaipal-Jamani and Angeli, 2017). Sequencing is the ability to put actions in the correct order (Kazakoff et al., 2013). Flow control verifies that each instructional step is sequenced correctly to ensure the activity runs flawlessly. When writing an algorithm, each step of a solution must be defined in detail and in the correct order. Therefore, sequencing and flow control are important cognitive processes to create algorithms without errors (Angeli et al., 2016). An algorithm can be evaluated by executing and monitoring it for potential errors. The debugging process identifies and eliminates these potential errors (Hsu et al., 2018). Generalization fosters flexible computational problem-solving by transferring devised solutions to other tasks with the same properties. Algorithms addressing subproblems can then be used synergistically and thus simplify future problem-solving processes (Curzon et al., 2014). Even though these processes are tailored very specifically to the design of algorithms in problem solving, they can also be seen more generally as a skill set relevant for problem solving or scientific inquiry (Shute, 1991; Bers, 2018). Nevertheless, the ability to solve problems by writing a program is typically viewed as very central to CT (Román-González et al., 2017; Shute et al., 2017). Acquiring and training CT skills as early as in primary school–usually using simple programming tools–may foster problem solving skills, cognitive flexibility and metacognition, which are main goals of primary education (National Research Council, 2010; Di Lieto et al., 2017; OECD, 2018). Furthermore, CT as a part of STEM education can be implemented at an early stage, maybe even serving as a motivator to pursue STEM when growing up (Shute, 1991; Nugent et al., 2015). Thus, preservice teachers need to understand CT and its importance and learn about possibilities to implement CT in primary school.

When teaching CT to preservice teachers, the complex subject of programming can be simplified by implementing low-threshold tasks (Weber et al., 2021). Low-threshold tasks can be short and simple programming exercises in a context that is familiar to preservice teachers and easy to implement into their future classrooms (e.g., a bicycle light, a traffic light, a metronome). Furthermore, low-threshold tasks may lower the preservice teachers’ anxiety of failure and their reluctance to program (Bescherer and Fest, 2019). Nevertheless, CT skills can also be acquired using logical reasoning or means-ends analyses and do not necessarily have to be programming tasks (Zhang and Nouri, 2019). However, using robots and computers may additionally motivate preservice teachers to learn CT, because they might expect their future students to have a fun experience by programming robots and computers (Garet et al., 2001).

### The present investigation

Even though the teaching of CT is a goal for educational policy (Ministerium für Bildung, Wissenschaft, Jugend und Kultur, 2018), CT is severely neglected in German primary schools (Feierabend et al., 2019). Studies suggest that introducing CT in primary educational levels is beneficial for developing problem-solving skills as a part of the 21st century skills (Jaipal-Jamani and Angeli, 2017; Yadav et al., 2017; Lamprou and Repenning, 2018). As has been shown in science education in primary school, high emotional costs and uncertainty about teaching CT may discourage teachers from integrating this important learning objective into their classrooms (Jaipal-Jamani and Angeli, 2017). To address these reservations, we implemented a university seminar to educate preservice teachers in CT, while addressing their expectancies and values towards this learning objective to motivate and increase their readiness to implement CT into their future classrooms. According to the literature, both components can be fostered with interventions that target expectancies and values (Wigfield et al., 2017). The literature suggests six characteristics that should be fulfilled to foster expectancies and values as stated above. (1) Interventions should have content goals; (2) preservice teachers should have control over tasks; (3) interventions should include hands-on-activities; (4) interesting tasks should be implemented; (5) collaborations between preservice teachers should be allowed, and (6) lecturers should emphasize the importance of the learning objective.

Due to the Covid-19 pandemic, the 11-week seminar was conducted online.

In this context, we specify the following research questions:

Can instruction in the context of this seminar

1. Improve preservice teachers’ expectancies of success—i.e., their self-concepts and self-efficacy?
2. Improve the value preservice teachers attribute to programming—i.e., intrinsic value, utility value, and attainment value?
3. Decrease preservice teachers’ programming anxiety, i.e., their emotional costs?
4. Are these changes dependent on preservice teachers’ prior CT competences?

## Materials and methods

### Participants

In total, 311 German university students participated in the study, of which 153 (133 female, 20 male) studied M.Ed. primary school education and attended a science education seminar on learning and teaching CT and programming to primary school children (experimental group, EG). The students were on average *M* = 24.32 years old, *SD* = 2.67, and in their *M* = 1.39, *SD* = 1.39 master semester, range = 1–12.

The remaining 158 participants (128 female, 30 male) studied M.Ed. special education at the same university and did not attend the science education seminar (baseline group, BG). The BG was implemented to detect possible retest effects. On average, the students were *M* = 24.47 years old, *SD* = 2.61, and in their *M* = 2.41, *SD* = 1.49, master semester, range = 1–10.

In Germany, primary school preservice teachers take courses in German, a foreign language (English or French), mathematics, and science throughout their 6-semester B.Ed. as well as 2-semester M.Ed. programs. These courses focus on both the domains and the teaching of domain-specific contents to primary school children. Special education preservice teachers study subject-related courses such as German, foreign languages, mathematics, or science without a focus on teaching these subjects during the first four semesters of their B. Ed. studies. The fifth and sixth semester as well as their 2-semester master program focus on special education needs and pedagogy, while subject-related courses are not implemented.

All students were informed about the goal of the study and gave their written consent to participation.

### Procedure

The EG attended an online science seminar targeting CT, including the use of a block-based programming language and teaching CT in primary school (see Supplementary Material 1 for the seminar plan). The seminar aimed at enhancing preservice teachers’ expectancies of success in programming and teaching CT, to reduce their programming anxiety, and to teach them why it is an important topic for primary school children (value components).

The seminar concept was designed by the authors following the six characteristics in the framework suggested by Wigfield et al. (2017). We addressed these characteristics as follows. (1) The seminar had the *content goal* of learning low-threshold programming skills and how to teach CT to primary school children. For every task the preservice teachers had to complete, they received explanations on why the task served that content goal to help students structure their learning and provide them with a sense of purpose (van de Pol et al., 2010; Belland et al., 2013). For example, the lecturer explained why and how a task supported students’ programming competences. (2) Preservice teachers needed to complete all tasks to pass the seminar. However, as the seminar was asynchronous, preservice teachers had *control* over when they wanted to work on the tasks and what resources they used. Control was provided to support students’ desire for autonomy and prevent them from getting frustrated (Wigfield and Tonks, 2004; van de Pol et al., 2010). (3) The preservice teachers solved ten *hands-on low-threshold programming tasks* that are also suited for primary school students and could be implemented into their classrooms. This might have helped preservice teachers to realize the value of the learning content for their later career, as CT can be implemented into classrooms even with young learners (Angeli et al., 2016; Sengupta et al., 2018; Weber et al., 2021). (4) Tasks were made *interesting* through relating the programming tasks to everyday activities, such as programming a bicycle light or a dice. Throughout the course, it was also explained to the preservice teacher students that these tasks could be implemented directly into future classrooms, which aimed at increasing students’ interests (Garet et al., 2001). Moreover, the texts they had to read related to ways of implementing these tasks into their future classrooms (Angeli et al., 2016; Reuter et al., 2021). (5) Preservice teachers were encouraged to *support each other* during programming and have conversations about the learning content through an online forum implemented into the course. From the beginning of the semester, the preservice teachers were invited to ask all their questions *via* this online forum. Moreover, an online tutorial led by the lecturer allowed students to ask their questions directly to the lecturer and talk to their fellow students (Kong et al., 2018). (6) The lecturer *emphasized the importance of CT* for young children and highlighted that preservice teachers needed to learn it as well (Yadav et al., 2014). Moreover, the students were encouraged to think about reasons why CT might be important for their future profession or their everyday lives to foster their autonomy (Wigfield et al., 2017). This was supported by texts concerned with possibilities to teach CT to young learners.

The seminar was piloted in a prior study focusing on programming anxiety (Weber et al., 2021). Moreover, students in the pilot cohort were asked to evaluate the seminar. Following this procedure, the seminar was slightly altered. First, the theoretical input was altered to focus stronger on scaffolding CT skills. Second, the students received a low-threshold introduction to CT and programming. The students were provided more time to familiarize themselves with the theoretical foundations of CT and received simple logic tasks before starting on the programming tasks.

**Figure 1:**
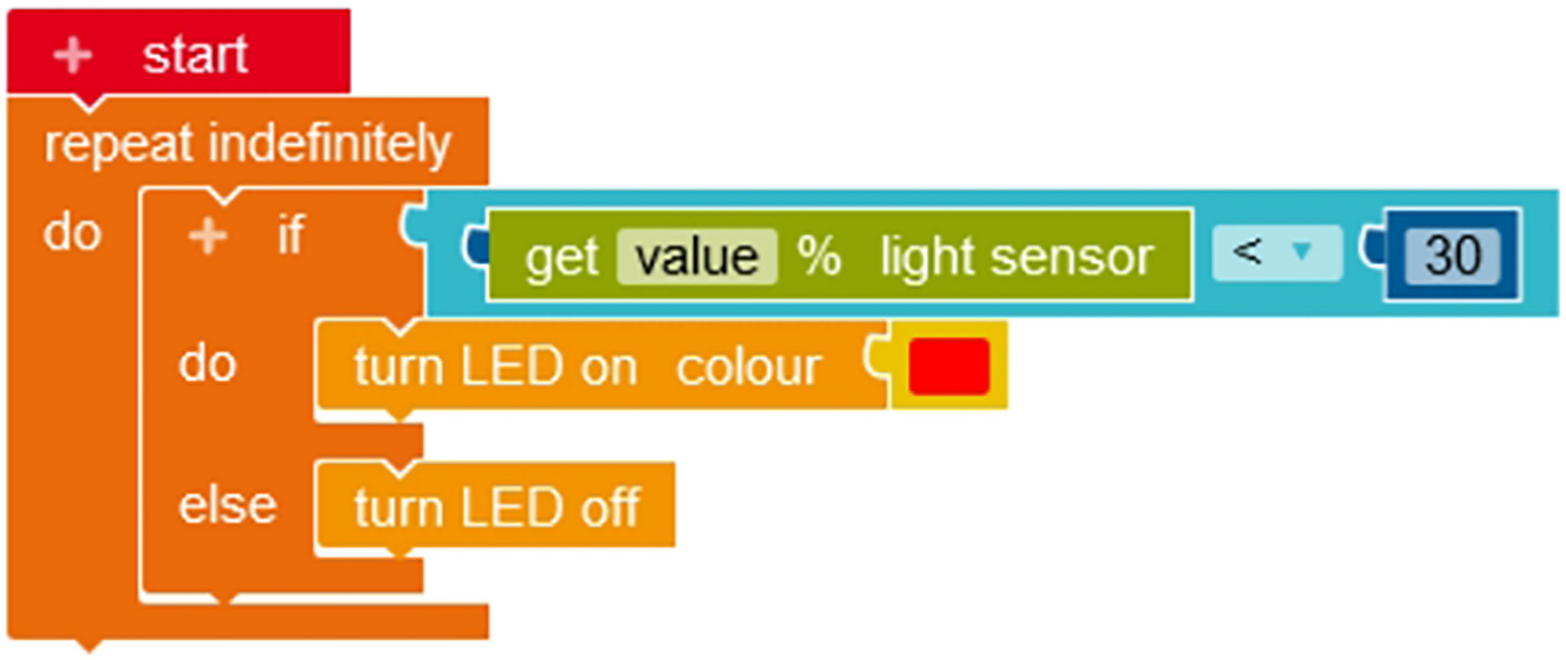
Example program for Calliope Mini^®^ using the NEPO^®^ programming language. Reproduced with permission from Open Roberta Lab, Fraunhofer-Institut für Intelligente Analyse-und Informationssysteme IAIS.

Programming was taught with problems that could be solved with Calliope Mini^®^, a microcontroller developed in Germany for primary school children. This controller can be programmed using the block-based programming language NEPO^®^. In such block-based programming languages, contrary to text-based programming languages, the commands are clustered in blocks that can be matched to form a program, so that codes do not need to be typed out which minimizes potential sources of errors. The microcontroller has multiple features, including different sensors for light, compass, sound, and temperature, can play tunes, and has multiple LED lights. To use Calliope Mini^®^, a tablet, computer or another mobile device is necessary. The programming platform Open Roberta Lab^1^ can be used for programming. An example program for one of the programming tasks of the seminar (program a bicycle light that lights up automatically when it gets dark) is presented in Figure 1 (for all programming tasks implemented in the seminar and their correct solutions, please see Supplementary Material 2).

To program the bicycle light that lights up automatically in darkness, the following steps need to be taken:

1. Determine value of light sensor.
2. Turn on the lamp if this value is below 30 (if-do-else, <30), otherwise turn off the lamp.

These two steps must be executed repeatedly and indefinitely. Thus, the students needed to program a loop, an if-then-else condition, and an action command.

In the seminar, the preservice teachers first received theoretical explanations about CT and an introduction into programming with Calliope Mini®. For this, the preservice teachers were first left to explore the programming environment by themselves for as long as they liked. Then they received a video with explanations and step-by-step programming instructions. Students were free to pause or rewind the video at any given time.

The lecturer explained why CT is an important competence for primary school children that they can acquire through low-threshold programming tasks. She also informed the students that studies demonstrated that many preservice teachers are skeptical or fearful of programming. She explained that one goal of the seminar was to alleviate such inhibitions. Moreover, the preservice teachers received explanations on the relevance of each assignment for programming and the teaching of CT. During the course of the semester, the preservice teachers solved ten programming assignments to foster their CT, such as programming a dice, a bicycle lamp, and a metronome. The students each handed in a solution for the programming task in the form of a screenshot. They were free to collaborate on the assignments *via* the provided online forum or through other means of their own choice.

The students received different forms of feedback during the seminar. After handing in their solutions, sample solutions for the programming tasks were provided. Moreover, students could ask questions in the online forum to receive feedback by the peers or ask the lecturer for help. Additionally, the lecturer was available *via* email, online during her office hours as well as during a voluntary online tutorial in which she met with students to discuss their questions about programming and the other seminar topics.

By implementing this seminar, we aimed to investigate whether learning to program with easy assignments and how to teach CT through programming to primary school children could change preservice teachers’ expectancies and values towards programming.

The BG did not receive any formal introduction into programming or CT, but only filled out the survey concerned with EVT and solved the CT tasks.

### Measures

All EVT measures were administered as pre-and posttests at the beginning and the end of the 11-week course for the EG. For the BG, the measures were administered as pre-and posttests at a four-week interval. The items for the EVT measures were adapted from the PISA items to measure participants’ expectancies, values and anxiety towards programming following Marsh et al. (2019). Each item was rated on a 4-point Likert scale from strongly agree to strongly disagree. All EVT scales were tested for their structure with confirmatory factor analyses (CFAs) with the robust Yuan-Bentler correction. The results showed that all items loaded significantly on their corresponding factor, all standardized loadings > 0.40, all *p* < 0.001 (Matsunaga, 2010; Eid et al., 2015). Moreover, overall all CFAs showed satisfactory fits (Eid et al., 2015). For more information on the CFAs, please refer to the supplementary materials (Supplementary Material 3).

#### Programming expectancies

Programming self-concept focused on programming abilities (Eccles and Wigfield, 1995), and was measured with five items, e.g., *I learn programming quickly*. Cronbach’s α for programming self-concept was α = 0.75 at pretest and α = 0.88 at posttest. Programming self-efficacy relied on Bandura’s (1977) conceptualization of self-efficacy as self-confidence in specific tasks. It was measured with four items, of which each concerned a different programming task, e.g., *I think I can program loops*. Cronbach’s α for programming self-efficacy was α = 0.90 at pretest and α = 0.91 at posttest.

#### Programming values

Intrinsic value for programming was construed as the enjoyment of programming (Wigfield et al., 1997; Eccles, 2009), and measured with five items, e.g., *I enjoy programming*. Cronbach’s α for intrinsic value was α = 0.85 at pretest and α = 0.92 at posttest. Moreover, programming utility value measured the value preservice teachers placed on learning to program in relation to their later work as (science) teachers, e.g., *Learning to program is worthwhile for me because I will teach it to children later*. Cronbach’s α for programming utility value was α = 0.87 at pretest and α = 0.91 at posttest. Programming attainment value was conceptualized as preservice teachers’ perceived value of children learning to program and was measured with four items, e.g., *It is important to me that children understand basic algorithms*. Cronbach’s α for programming attainment value was α = 0.84 at pretest and α = 0.85 at posttest. The fourth value component, emotional costs, was conceptualized as programming anxiety following Wigfield and Meece’s (1988) conceptualization of mathematics anxiety as feelings of worry, stress, and helplessness. It was assessed with six items, e.g., *I worry that it will be difficult for me to solve programming tasks*. Cronbach’s α for programming anxiety was α = 0.84 at pretest and α = 0.85 at posttest.

#### Computational thinking

**Figure 2:**
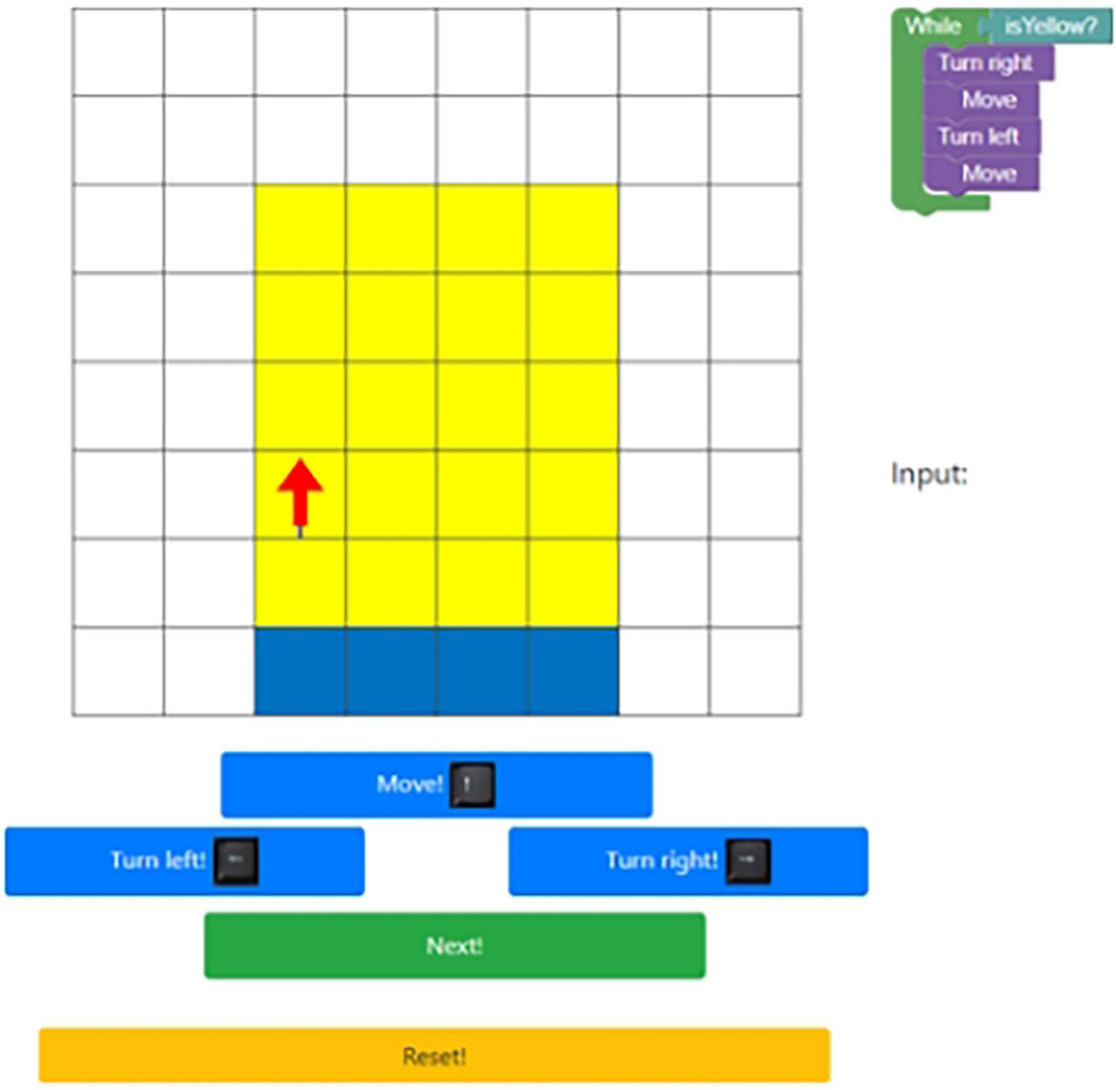
Presentation of test environment for an item in Progly.

CT was assessed with Progly (Bastian et al., 2021) through programming tasks, which is in line with other studies on CT (Román-González et al., 2017; Shute et al., 2017). Progly is a test system in which the preservice teachers need to solve tracing tasks. It presents the preservice teachers with nine different items which were adapted from a psychometric test developed by Mühling et al. (2015), and validated by Bastian et al. (2021). For each item the test system presents the preservice teachers with an 8 × 8 grid, a figure in the grid, a program snippet and control buttons to move the figure in the grid. The order of the items is presented in Table 1 and an example for one of the items is presented in Figure 2.

**Table 1:** Order of the Progly items measuring CT.

Item	Description
1	Sequence of simple commands
2	Repetition with a fixed number of iterations
3	Two nested repetitions with a fixed number of iterations
4	Repetition with an exit condition
5	Conditional command with a true condition
6	Conditional command with a false condition
7	Conditional command with a repetition with a fixed number of iterations
8	Repetition with a fixed number of iterations in a repetition with an exit condition
9	Repetition with an exit condition in a repetition with a fixed number of iterations

The test assesses the ability to execute common computational concepts like sequences, loops (with and without conditions) or conditional statements (Brennan and Resnick, 2012). During the measurement every interaction with the test system is recorded.

The task for each item is to move the figure exactly like the presented code would move it. The answer, thus, is a sequence of steps. An item is marked as correct if the sequence matches the single correct sequence for the presented code. The test score is the sum of correctly solved items out of the nine items presented. After the measurement the preservice teachers received feedback on how many items they had solved correctly based on the global score. Cronbach’s α for CT was α = 0.75.

### Data analysis

The statistics program R, version 4.2.0 (R Core Team, 2022), was used for data analyses. We used the psych package (Revelle, 2021) for descriptive and correlation analyses, and the lme4 (Bates et al., 2015) and lmerTest packages (Kuznetsova et al., 2017) for the specification of mixed effects models. To investigate the change in the EVT components as well as group differences between the EG and the BG depending on prior CT, we specified mixed-effects models with person on level-2 and time of measurement on level-1.

## Results

The descriptive statistics are presented in Table 2 and the correlations between the constructs are presented in Table 3. The descriptive statistics indicate that both the expectancy as well as the value components increased from pretest to posttest in both groups, while programming anxiety decreased. In the EG, self-concept and self-efficacy showed large increases, while the increase in intrinsic value, utility value and attainment value were smaller. Moreover, programming anxiety showed a rather large decrease from pre-to posttest. A similar trend can be observed in the BG. However, increase in self-concept, self-efficacy and intrinsic value was descriptively smaller than in the EG. Moreover, there was no increase in utility value and attainment value and the decrease in programming anxiety was again smaller than in the EG. A *t*-test implied that CT did not differ between the two groups, *t*(308.77) = 0.45, *p* = 0.653.

**Table 2:** Descriptive statistics by condition and point of measurement.

	Experimental group					Baseline group
	Pretest			Posttest		Pretest	Posttest		
	*n*	*M*	*SD*	*n*	*M*	*SD*	*n*	*M*	*SD*	*n*	*M*	*SD*
SC	152	0.70	0.50	153	1.50	0.49	158	0.50	0.46	158	0.64	0.47
SE	152	0.69	0.66	153	1.58	0.57	158	0.22	0.42	158	0.22	0.39
IV	152	1.34	0.60	153	1.51	0.71	158	0.78	0.55	158	0.87	0.62
UV	152	1.44	0.67	153	1.61	0.61	158	0.66	0.57	158	0.66	0.62
AV	152	1.72	0.61	153	1.87	0.59	158	1.61	0.67	158	1.59	0.69
PA	152	1.73	0.77	153	1.29	0.67	158	1.83	0.66	158	1.68	0.68
CT	153	3.71	2.29	–	–	–	158	3.59	2.30	–	–	–

SC, Self-concept. SE, Self-efficacy. IV, Intrinsic value. UV, Utility value. AV, Attainment value. PA, Programming anxiety. CT, Computational thinking.

**Table 3:** Correlations of the constructs.

	SC Pre	SE Pre	IV Pre	UV Pre	AV Pre	PA Pre	CT	SC Post	SE Post	IV Post	UV Post	AV Post
SE Pre	0.62^***^											
IV Pre	0.54^***^	0.44^***^										
UV Pre	0.32^***^	0.41^***^	0.65^***^									
AV Pre	0.20^***^	0.24^***^	0.47^***^	0.57^***^								
PA Pre	−0.48^***^	−0.26^***^	−0.37^***^	−0.18^**^	−0.03							
CT	0.09	0.03	0.13^*^	−0.01	0.09	−0.13^*^						
SC Post	0.46^***^	0.43^***^	0.62^***^	0.49^***^	0.21^***^	−0.30^***^	0.12^*^					
SE Post	0.35^***^	0.48^***^	0.52^***^	0.53^***^	0.19^**^	−0.18**	0.07	0.80^***^				
IV Post	0.37^***^	0.32^***^	0.67^***^	0.44^***^	0.33^***^	−0.22^***^	0.20^***^	0.75^***^	0.61^***^			
UV Post	0.28^***^	0.35^***^	0.55^***^	0.65^***^	0.42^***^	−0.09	0.13^*^	0.65^***^	0.72^***^	0.68^***^		
AV Post	0.17^**^	0.15^**^	0.39^***^	0.38^***^	0.65^***^	−0.09	0.14^*^	0.39^***^	0.38^***^	0.50^***^	0.63^***^	
PA Post	−0.39^***^	−0.29^***^	−0.37^***^	−0.21^***^	−0.09	0.50^***^	−0.18^**^	−0.57^***^	−0.43^***^	−0.46^***^	−0.32^***^	−0.16^**^

SC, Self-concept. SE, Self-efficacy. IV, Intrinsic value. UV, Utility value. AV, Attainment value. PA, Programming anxiety. PE, Prior experience with programming. CT, Computational thinking;

* *p* < 0.05;

** *p* < 0.01;

*** *p* < 0.001.

Moreover, the correlations imply that the expectancy and value components are positively intercorrelated between the two measurement points. This indicates that higher expectancies of success, i.e., a higher self-concept and self-efficacy, are related to higher values as well, i.e., intrinsic, utility and attainment value. Programming anxiety is negatively correlated with the other constructs. CT is negatively correlated with programming anxiety at pretest. This indicates that preservice teachers with high prior CT skills are less likely to be anxious about programming. Furthermore, CT is positively correlated with intrinsic value at pretest. This implies that preservice teachers with high CT are more likely to enjoy programming before taking part in the seminar. CT was positively correlated with self-concept, intrinsic, utility and attainment value, and negatively correlated with programming anxiety at posttest.

To answer the research questions, we examined group differences in change. Differences between participants as indicated by the intraclass correlations explained 16% of variance in self-concept, 23% in self-efficacy, 60% in intrinsic value, 60% in utility value, 63% in attainment value, and 41% in programming anxiety. This indicates that the points of measurement are nested in persons. Thus, we specified mixed-effects models with preservice teachers on level-2 and included time on level-1. The results are presented in Table 4.

**Table 4:** Mixed level models.

	Self-concept			Self-efficacy			Intrinsic value			Utility value			Attainment value			Programming anxiety		
	*ρ*			*ρ*			*ρ*			*ρ*			*ρ*			*ρ*		
ICC	0.16			0.23			0.60			0.60			0.63			0.41		
Fixed effects	*γ*	*p*	*SE*	*γ*	*p*	*SE*	*γ*	*p*	*SE*	*γ*	*p*	*SE*	*γ*	*p*	*SE*	*γ*	*p*	*SE*
ΔIntercept BG–EG	0.19	0.000	0.05	0.47	0.000	0.06	0.55	0.000	0.07	0.76	0.000	0.07	0.11	0.132	0.07	−0.10	0.207	0.08
Time*BG	0.13	0.010	0.05	0.04	0.552	0.06	0.07	0.236	0.06	0.00	0.971	0.06	−0.04	0.515	0.05	−0.15	0.037	0.07
Time*EG	0.79	0.000	0.05	0.89	0.000	0.05	0.16	0.000	0.05	0.17	0.000	0.05	0.15	0.000	0.04	−0.44	0.000	0.05
ΔTime*BG–EG	0.66	0.000	0.07	0.85	0.000	0.08	0.10	0.208	0.08	0.17	0.044	0.08	0.19	0.009	0.07	−0.29	0.002	0.09
CT	0.02	0.017	0.01	0.02	0.143	0.01	0.04	0.001	0.01	0.01	0.253	0.01	0.03	0.017	0.01	−0.04	0.003	0.01
***Random effects***	***Var***	***SD***		***Var***	***SD***		***Var***	***SD***		***Var***	***SD***		***Var***	***SD***		***Var***	***SD***	
Person	0.09	0.30		0.07	0.27		0.19	0.44		0.16	0.41		0.25	0.50		0.23	0.48	
Level-1 Residuum	0.14	0.38		0.22	0.46		0.18	0.43		0.22	0.47		0.15	0.39		0.26	0.51	

Concerning research question 1 for the expectancy components, the EG had a higher self-concept as well as a higher self-efficacy than the BG at pretest. Moreover, in both groups self-concept increased from pretest to posttest, while self-efficacy only increased in the EG. The increase was higher in the EG compared to the BG for both self-concept and self-efficacy.

Research question 2 regarded the value components. The EG showed higher values on intrinsic value and utility value compared to the BG. However, for attainment value, there were no group differences between the EG and the BG at pretest, indicating that both groups viewed it as equally important for children to learn about programming. All three value components increased in the EG, but not in the BG. However, the difference in change between the two groups for intrinsic value was small and thus not significant.

For research question 3 concerned with programming anxiety, at pretest, both groups had equally high programming anxiety. It decreased in both groups from pretest to posttest and the decrease was larger in the EG compared to the BG.

Research question 4 regarded the effect of CT on the change in the EVT components. CT positively affected the change in preservice teachers’ self-concepts, intrinsic and attainment values. Moreover, it was negatively related to their programming anxiety, indicating that higher CT skills affected the decrease in emotional costs. These results suggest that higher prior CT skills are related to positive outcomes and underline the need for the implantation of CT into teacher education.

## Discussion

Supporting preservice teachers in acquiring CT skills and preparing them to teach CT to children is of importance (National Research Council, 2010; Yadav et al., 2014; OECD, 2018). This might be achieved through enhancing their expectancies and values regarding the subject and the teaching of the subject (Wigfield and Guthrie, 2010; Wigfield et al., 2017). However, studies on ways of implementing interventions into higher education that foster expectancies and values towards CT remain sparse.

Therefore, we conducted a study on whether a seminar with low-threshold programming tasks can foster preservice teachers’ expectancies of success and values towards teaching CT and programming to young learners. Moreover, we examined whether the seminar decreased their emotional costs, i.e., their programming anxiety. Thus, we compared primary school preservice teachers who attended a mandatory science seminar on CT and programming to special education preservice teachers who did not attend the science seminar and did not learn about CT or programming during their course of study. The results suggest that a science seminar implementing low-threshold programming tasks fostered preservice teachers’ expectancies and values towards CT and programming compared to the BG.

Our results implied that expectancies and values were interrelated at pretest as well as at posttest. Moreover, we found interrelations between the constructs between pretest and posttest. These relations are in line with previous research (e.g., Nagengast et al., 2011) and indicate that preservice teachers who expect to be successful in a domain tend to value this domain as well. Programming anxiety was negatively related to the other EVT components. This implies that higher emotional costs come at the expense of expectancies of success as well as values. Thus, preservice teachers who had higher anxiety towards programming, did not expect to do well and might have concluded that programming is not enjoyable or valuable for themselves or their future students (Lee et al., 2013).

For CT, results implied positive relations with self-concept, intrinsic value, utility value and attainment value and a negative relation with programming anxiety. This indicates that preservice teachers who had higher CT skills were more likely to have higher expectancies and values towards programming and were less anxious about it (Dowker et al., 2016; Marsh et al., 2019). Thus, competences were related to expectancies of success and values, as suggested by EVT (Eccles, 2009).

### Group differences at pretest

We found differences at pretest between the primary school preservice teachers who attended the seminar and the special education preservice teachers who did not. Primary school preservice teachers held higher expectancies of success in programming, i.e., self-concept and self-efficacy, and higher intrinsic and utility values towards programming than the special education preservice teachers. A reason for these differences might be that the seminar was a mandatory course and primary school preservice teachers concluded that they will need to teach CT later in their career (Yadav et al., 2014; Jaipal-Jamani and Angeli, 2017; Butler and Leahy, 2021). Thus, they might have deduced that it is important for them to learn it as well, which might explain the higher intrinsic value and utility value. Moreover, they might have assumed that they will be able to learn it at least to some degree, because the university designed and implemented a seminar for primary school preservice teachers. This implies that the university trusted in their abilities to learn CT and programming, which might explain their higher self-concepts and self-efficacy compared to the special education preservice teachers (Bouck et al., 2021; Faherty et al., 2021). However, primary school preservice teachers’ expectancies of success were rather low at the beginning of the semester. None of the participants had gained prior experiences with CT during their studies. This might explain why they viewed themselves as quite unable at programming as a central part of CT. On the other hand, the special education preservice teachers might believe that they will never need to teach CT to children themselves and thus do not value it for themselves and do not expect to be successful in programming (Jaipal-Jamani and Angeli, 2017; Bouck et al., 2021). In Germany, special education teachers mostly take a supporting role and focus on special education needs and pedagogy, while the primary school teachers will mainly be responsible for the teaching of the domain-specific subjects, in our case CT and programming.

However, the two groups did not differ in their attainment value and their emotional costs. This implies that both groups perceived programming as an important learning goal for primary school children and is in line with other studies (Bouck et al., 2021). Moreover, both had high emotional costs, suggesting that even though they think it is important for young children to learn about CT and programming, they are anxious about it themselves. This is in line with results on programming anxiety with computer science students (Nolan and Bergin, 2016) and might be partly explained by a stereotype threat, because most preservice teachers are women, who often tend to be anxious about science activities (Shapiro and Williams, 2012; Marsh et al., 2019).

The low expectancy beliefs, high values and emotional costs suggest that the students need support in CT and programming to prepare them to teach it to their future students. Therefore, the intervention in the form of a specifically designed seminar was called for.

### Change in the EVT components

Research questions 1–3 were concerned with changes in the EVT components during the seminar. The science seminar featured all six characteristics called for by Wigfield et al. (2017). Thus, the science seminar might have changed preservice teachers’ expectancies and values.

Expectancies and values increased in the EG, and this increase was higher than in the BG for all EVT components except intrinsic value. This is in line with literature on interventions that target EVT (Guthrie et al., 2004; Wigfield and Guthrie, 2010; Wigfield et al., 2017). Motivational components are often ignored at the university level, even though fostering expectancies and values is worthwhile, because motivational processes influence learning, plans and persistence (Bong et al., 2012; Lee et al., 2013). This study revealed that supporting expectancies and values can be achieved in an online seminar on CT and programming, which are particularly suitable for an online seminar.

For self-concept and programming anxiety, simply presenting preservice teachers with CT tasks in the form of programming such as Progly (Bastian et al., 2021), as in this study, caused changes for the special education preservice teachers. A reason might be that preservice teachers in the BG thought of programming as writing complex commands and codes. However, a short problem-solving task that involved a graphic programming language might have increased their beliefs that they could solve programming tasks and decreased their anxiety, because they realized that coding is just one part of CT and programming.

This poses the question why self-concept increased in the BG, but self-efficacy did not. Self-concept is a broader construct than self-efficacy (Trautwein et al., 2013). The CT task used in this study is based on programming (Román-González et al., 2017; Shute et al., 2017), but the special education preservice teachers did not code themselves. Thus, it might have increased their self-concepts, i.e., their expectancy that they are at least a little able at programming, but not their more specific self-efficacy that they can solve specific programming tasks such as being able to program loops. To foster self-efficacy beliefs about programming, these beliefs probably need to be addressed more specifically through programming tasks the preservice teachers solve themselves such as implemented in the seminar. This might be the reason that self-efficacy increased in the EG but not the BG. Nevertheless, both self-concept as well as programming anxiety changes were higher for the EG that visited the science seminar and gained more experiences with programming tasks (O'Mara et al., 2006; Dowker et al., 2016).

### Relations with CT

The fourth research question was concerned with the relation of CT with the EVT components. Results implied that CT was positively related with changes in self-concept, intrinsic and attainment value, but negatively related with changes in programming anxiety. As preservice teachers gained experiences with programming during the seminar, their changes in self-concepts probably reflected their abilities more correctly, because they realized that they were able to write low-threshold programs (Nagengast et al., 2011; Marsh et al., 2019). Moreover, CT was related to change in intrinsic value. This indicates that preservice teachers who enjoy programming are more likely to have gained experiences with it and students who are better at CT likely enjoy programming more (Hidi et al., 2017). Furthermore, preservice teachers who had higher CT skills might have had higher confidence in children’s ability to learn CT and programming. Thus, they might have become more open to the idea of children learning CT (Lee et al., 2013).

For the emotional costs, preservice teachers with higher CT were probably less anxious, because they might have been more familiar with problem-solving activities, which programming and CT are a part of (Wing, 2006; Schmader et al., 2008). Thus, higher CT might have increased students’ programming enjoyment and decreased their programming anxiety by providing them with confidence about their competences (Lee et al., 2013).

Concluding, the science seminar fostered expectancy and value components and decreased emotional costs for primary school preservice teachers through specifically tailored activities according to the six characteristics suggested by Wigfield et al. (2017).

### Limitations

The limitations of this study concern the implementation of the seminar as well as the implementation of the measurement.

The science seminar for the primary preservice teachers was conducted as an online seminar, because of the Covid-19 pandemic. However, implementing the seminar as an attendance seminar might have an advantage over an online course. In an attendance seminar, the lecturer would be able to cater to the preservice teachers’ needs while the students program and could apply support measures to help the preservice teachers learn. Thus, preservice teachers would be able to learn and experience the support measures they could use with children in their own future classrooms. The effect of an online seminar on the EVT components should thus be compared to an attendance seminar in a future study.

The limitations concerning the measures regard (1) that we measured CT only at pretest and (2) that the EVT components were only assessed at two points of measurement. (1) Our goal was to create and implement a science seminar that would foster the EVT components for CT. However, it would be interesting to investigate whether the seminar fostered CT at the same time and how a possible change in CT relates to the EVT component (Eccles, 2009). Thus, change in CT and its relation with expectancies and values could be investigated in a future study. (2) Moreover, it might be worthwhile to implement more points of measurement for the EVT components during the seminar to investigate changes over time. By doing that, it could be investigated at what point in the seminar the EVT components change the most and if this change is linked to certain components proposed by Wigfield et al. (2017).

Our baseline group consisted of M.Ed. special education preservice teachers. The reason for this decision was that the seminar on CT and programming was mandatory and thus, all M.Ed. primary school preservice teachers had to take it. Therefore, it was impossible to recruit primary school preservice teachers in their master programme as a baseline group. Thus, it remains unclear whether there might be a systematic difference between special education and primary school preservice teachers due to their course of study or because the special education preservice teachers thought that they would never have to teach CT or programming themselves. To investigate this further, the seminar could be made available as special credit for special education preservice teachers. However, the preservice teachers willing to participate in this seminar would probably be the ones with the highest motivation to learn CT and programming. Therefore, they would not be comparable to the primary school preservice teachers who have to attend the seminar.

We chose to target expectancies and values concerning teaching CT and programming through interventions that have content goals; give students control over tasks; include hands-on-activities; interesting tasks; allow collaborations between students; and emphasize the importance of the learning objective. However, it remains unclear whether the characteristics Wigfield and his colleagues (Guthrie et al., 2004; Wigfield and Guthrie, 2010; Wigfield et al., 2017) suggest are even relevant for preservice teachers’ expectancies and values towards CT and programming. Maybe primary school preservice teachers’ knowledge that they will have to teach CT eventually (Yadav et al., 2014) was enough to change the EVT components. This could be investigated by implementing two additional groups in addition to the one that already exists. In all three groups, the lecturer could explain that the preservice teachers will have to teach CT and programming during their career and that it is important for young children. The first group would follow the suggestions by Wigfield et al. (2017) as implemented in the present study. The second group would receive the same programming tasks but would be left to work on them by themselves and the third group would just receive the information without the programming tasks. Thus, it could be investigated whether the sole knowledge, a combination of knowledge and programming or the specifically designed seminar change the EVT components. The need for more seminars is a methodological issue that we could not investigate in this study, as this methodological desideratum is not feasible for the third group that would just receive information. Moreover, concerning the second group that would receive information and tasks, such an approach is not desirable, because there are more engaging ways to teach CT and programming, e.g., the seminar in this study.

Nevertheless, our study shows that students’ expectancies and values can be fostered and their emotional costs decreased during a science seminar following the suggestions by Wigfield et al. (2017). This can be achieved by using low-threshold programming tasks that offer the students ways to engage with CT and prepare them to teach it to primary school children in their own classrooms.

## Data availability statement

The raw data supporting the conclusions of this article will be made available by the authors, without undue reservation.

## Ethics statement

Ethical review and approval was not required for the study on human participants in accordance with the local legislation and institutional requirements. The patients/participants provided their written informed consent to participate in this study.

## Author contributions

AW, MB, and ML contributed to the conception and the design of the study. AW organized the database, performed the statistical analysis, and wrote the first draft of the manuscript. AW, MB, VB, AM, and ML wrote sections of the manuscript. All authors contributed to manuscript revision, read, and approved the submitted version.

## Funding

This research was funded by the Deutsche Telekom Stiftung and the German Federal Ministry of Education and Research, Grant Number 01JA1605.

## Conflict of interest

The authors declare that the research was conducted in the absence of any commercial or financial relationships that could be construed as a potential conflict of interest.

## Publisher’s note

All claims expressed in this article are solely those of the authors and do not necessarily represent those of their affiliated organizations, or those of the publisher, the editors and the reviewers. Any product that may be evaluated in this article, or claim that may be made by its manufacturer, is not guaranteed or endorsed by the publisher.

## Footnotes

^1^ https://lab.open-roberta.org
