# Supplementary material for: Fostering preservice teachers’ expectancies and values towards computational thinking
Source: Front Psychol. 2022 Sep 28;13:987761. doi: 10.3389/fpsyg.2022.987761 (PMC9555240; doi:10.3389/fpsyg.2022.987761)
Supplement: Supplementary file 1 [file Data_Sheet_1.docx]

**Fostering preservice teachers’ expectancies and values towards computational thinking**

Anke M. Weber, Morten Bastian, Veronika Barkela, Andreas Mühling, & Miriam Leuchter

**Supplementary Materials**

[Supplementary Material 1. Seminar plan for the science seminar implemented in the experimental group.](#_Toc108003766)

[Supplementary Material 2. Programming tasks and their solutions implemented into the seminar.](#_Toc108003767)All Tasks reproduced with permission from Open Roberta Lab, Fraunhofer-Institut für Intelligente Analyse- und Informationssysteme IAIS

[Supplementary Material 3. Results of the confirmatory factor analyses for the expectancy and value components.](#_Toc108003768)

# Supplementary Material 1. Seminar plan for the science seminar implemented in the experimental group.

Supplementary Table 1

*Seminar plan of the science seminar*

| Week | Theoretical input | Assignments |
| --- | --- | --- |
| 1 | Welcome video | Questionnaire EVT  Progly |
| 2 | Teaching materials for Calliope | Logic quiz |
| 3 | Introduction into CT and programming (video) | 7 programming tasks |
| 4 | Read article on CT curriculum | Test about knowledge of CT |
| 5 | - | Programming task “temperature warning system” |
| 6 | Read articles on scaffolding | Test on scaffolding |
| 7 | Read article on scaffolding  How to apply scaffolding in CT and programming | Scaffolding tasks |
| 8 | How to apply scaffolding in CT and programming | Scaffolding task |
| 9 | How to apply scaffolding in CT and programming | Programming task “alarm system” |
| 10 | - | Programming task “metronome” |
| 11 | - | Questionnaire EVT  Progly |

Supplementary Material 2. Programming tasks and their solutions implemented into the seminar. All Tasks reproduced with permission from Open Roberta Lab, Fraunhofer-Institut für Intelligente Analyse- und Informationssysteme IAIS

Task 1: Program a smiley.


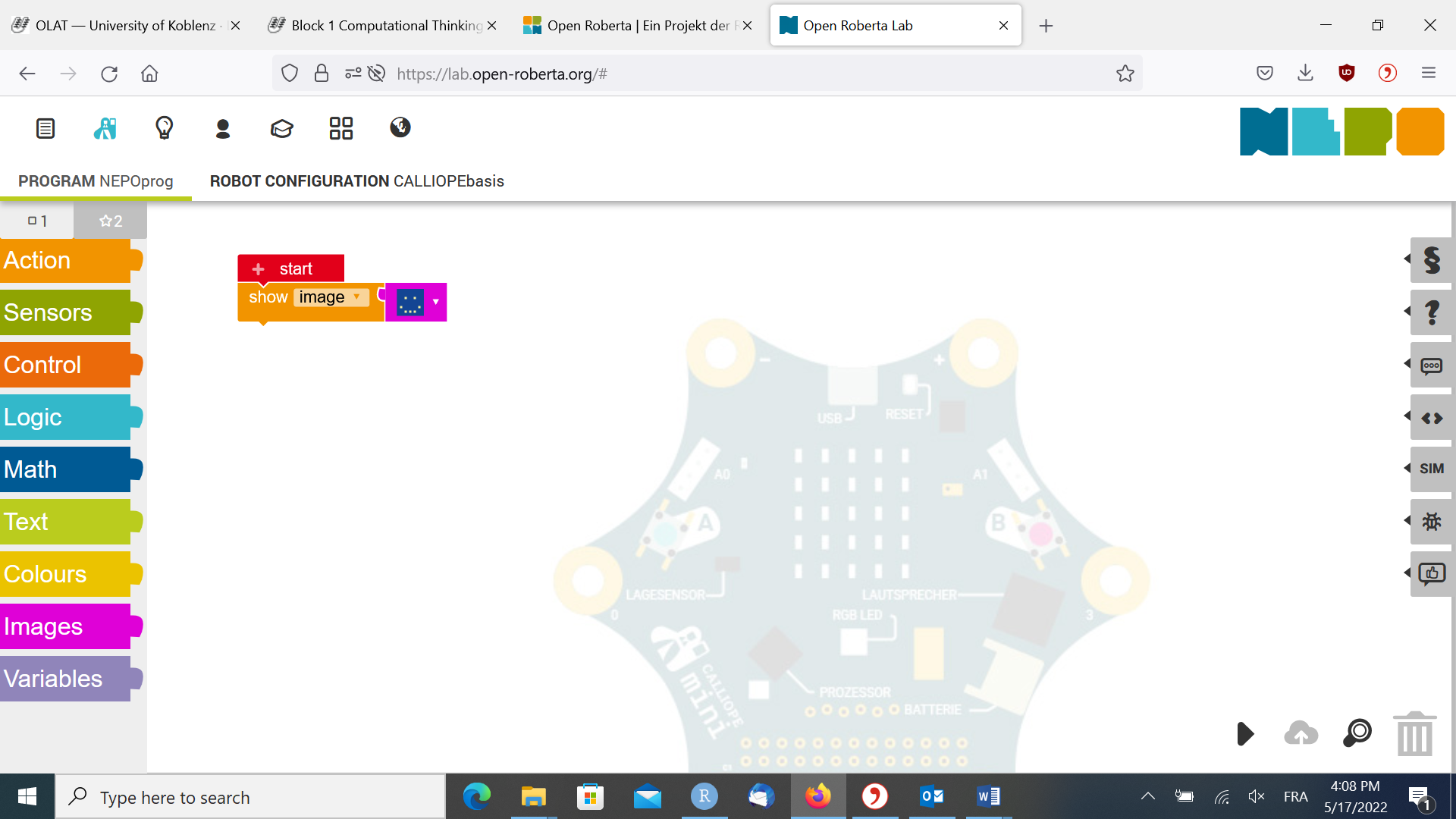


Task 2: Program an if-then-else command: If upside down, then show smiley, else show text *Hello*.


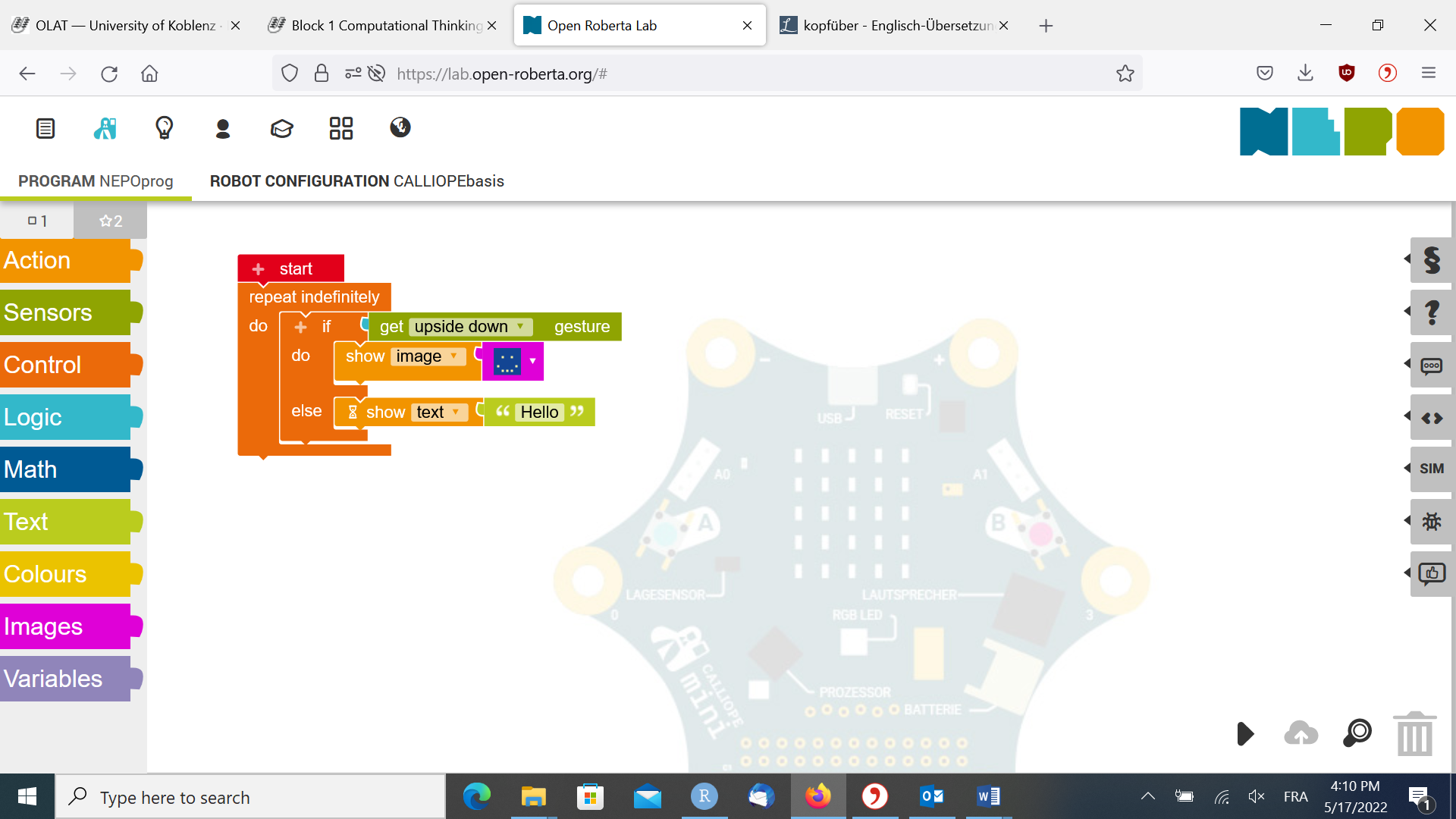


Task 3: Add conditions: If upside down and button A is pressed, then show text *Hello*.


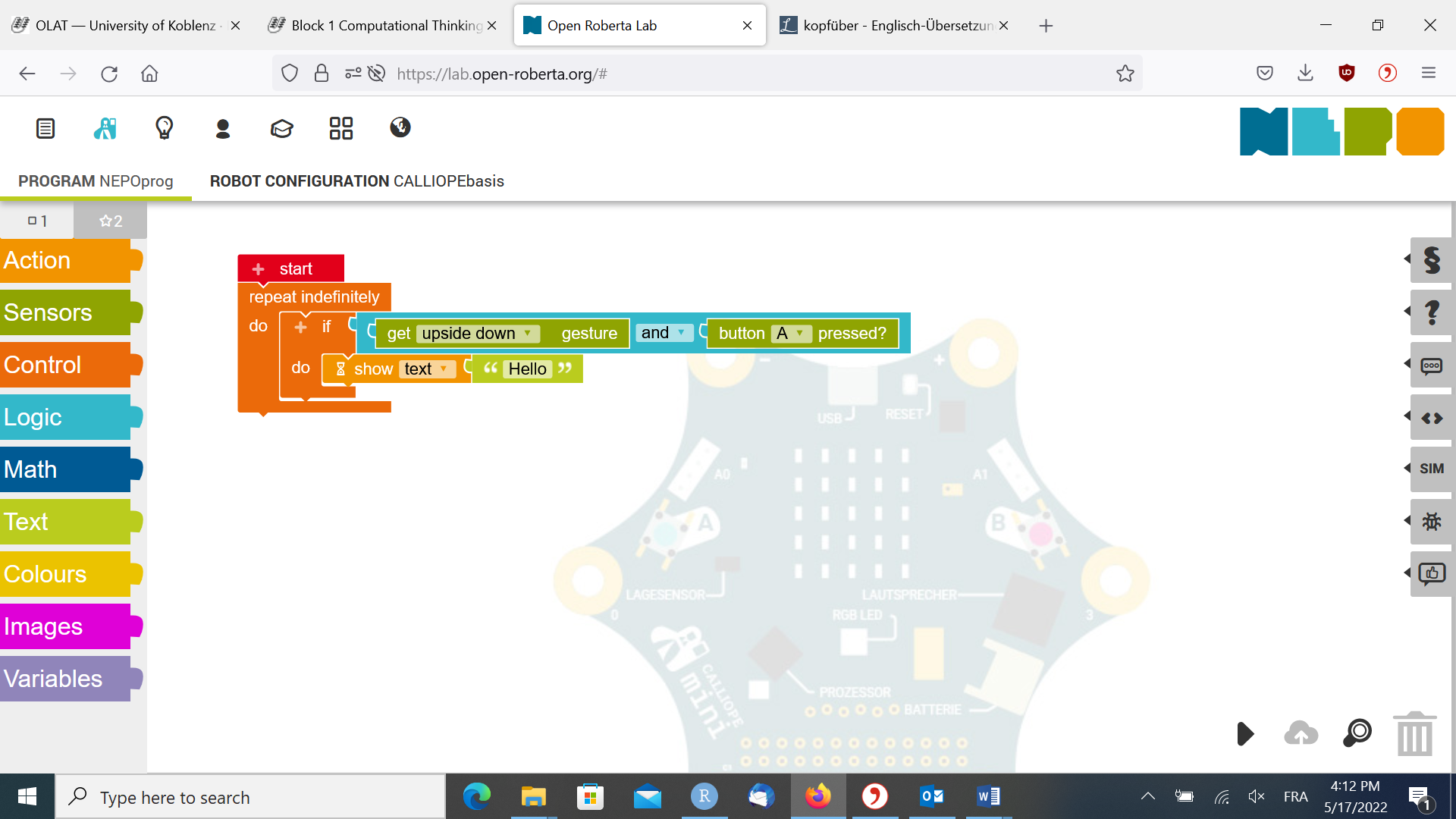


Task 4: Add conditions: If upside down and button A is pressed, then show text *Hello*. If only button A is pressed, then show a heart.


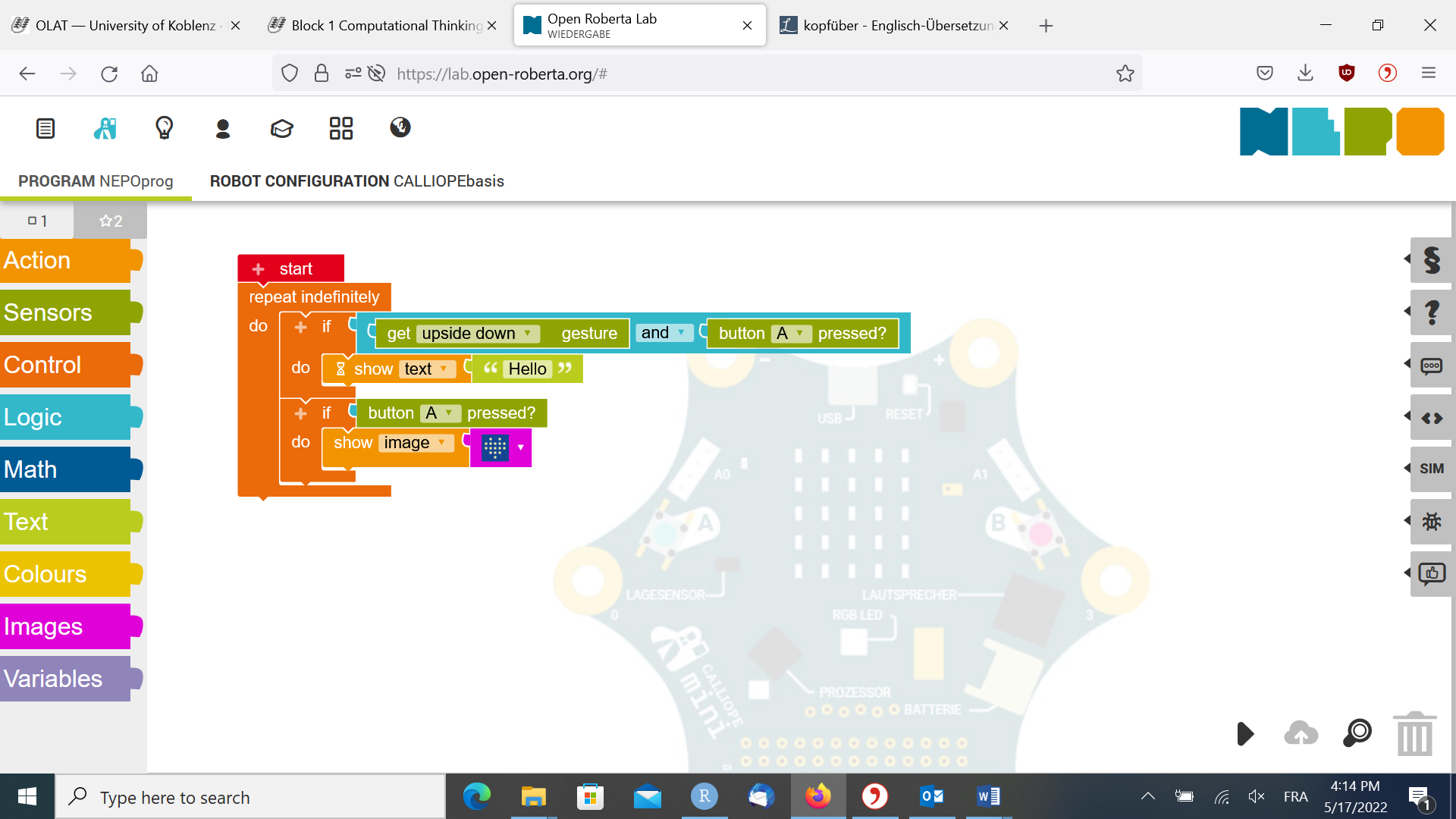


Task 5: Program a traffic light with a timer.


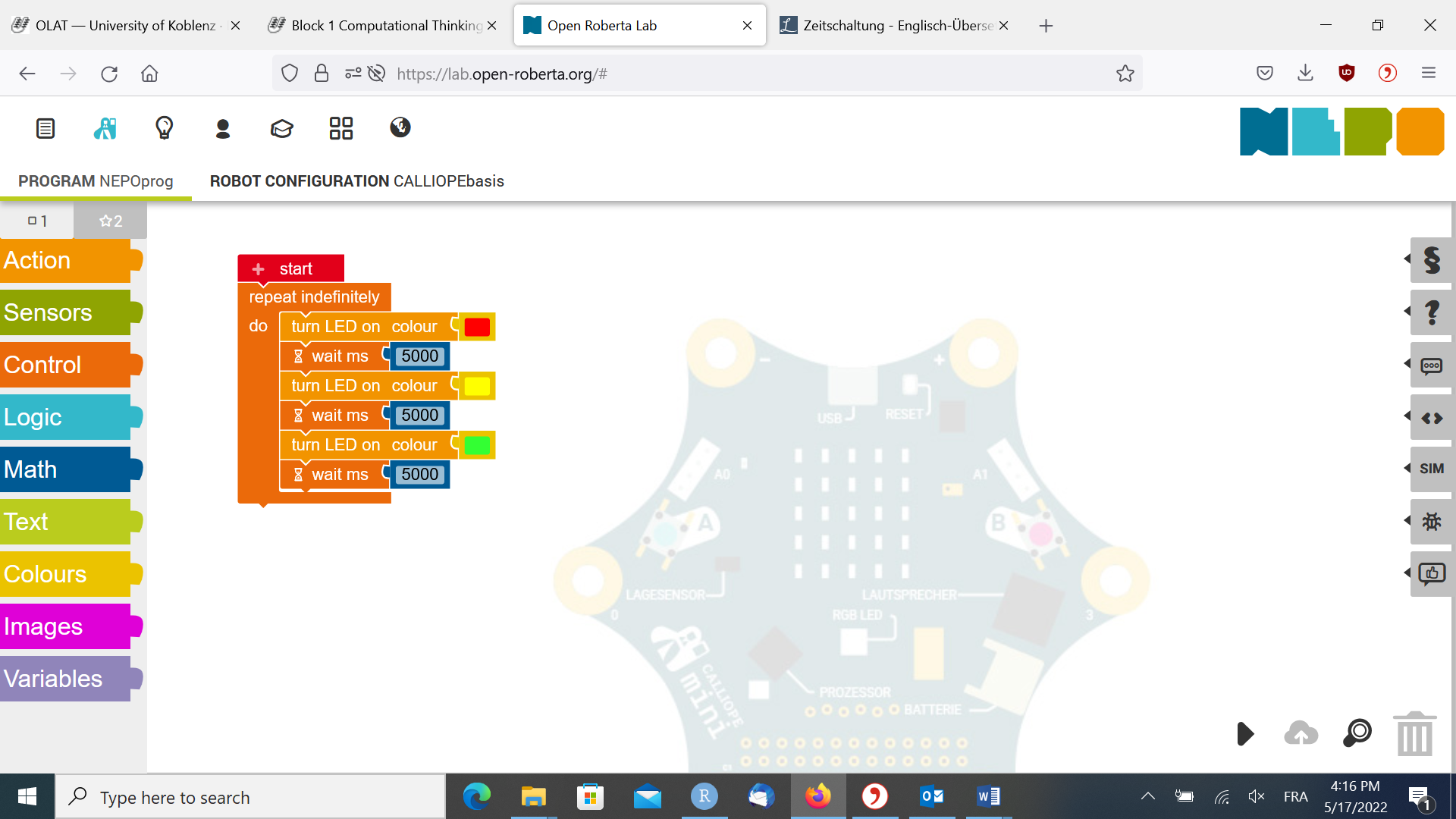


Task 6: Program a bicycle lamp that lights up automatically once it gets dark.


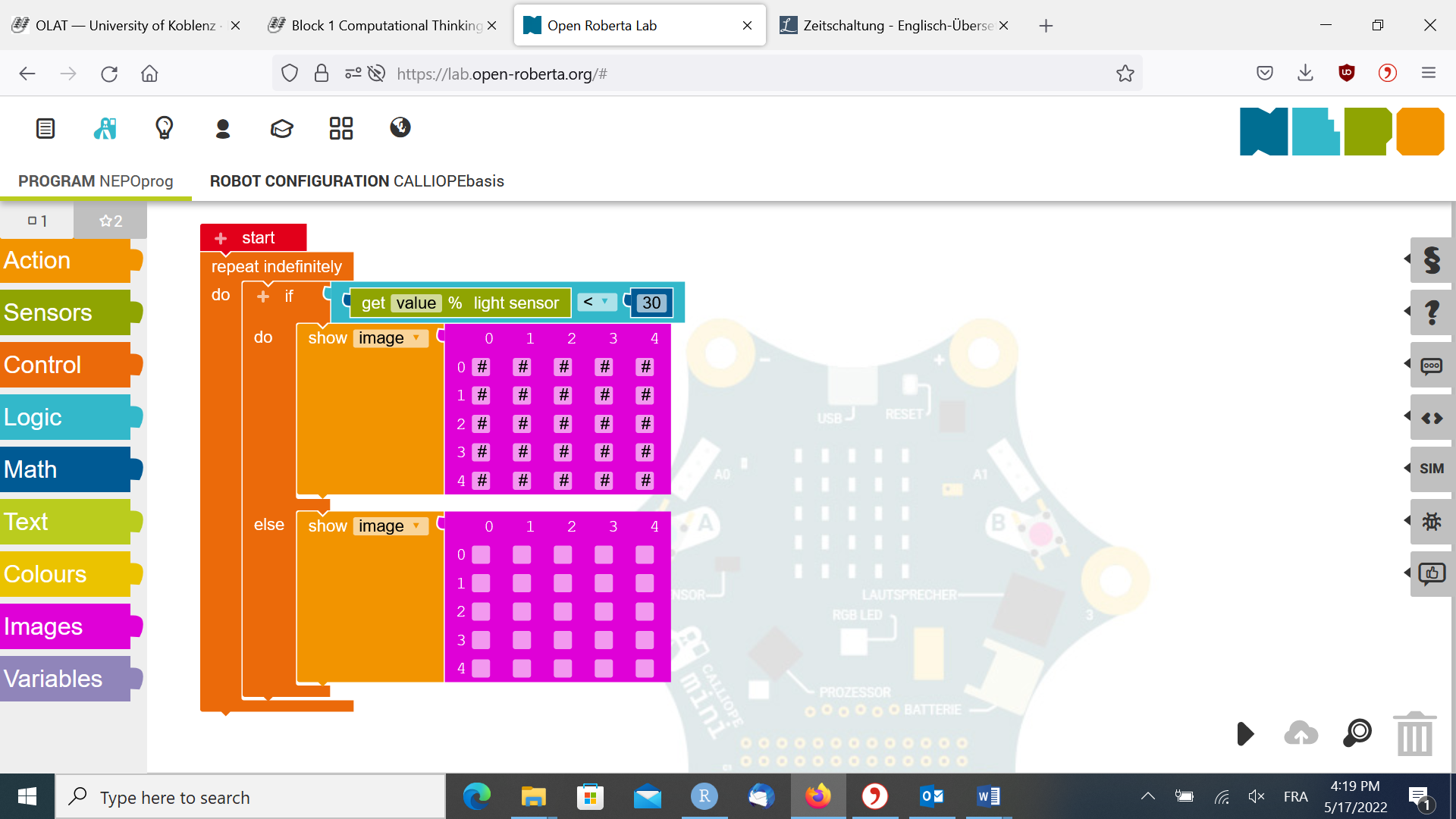


Or:


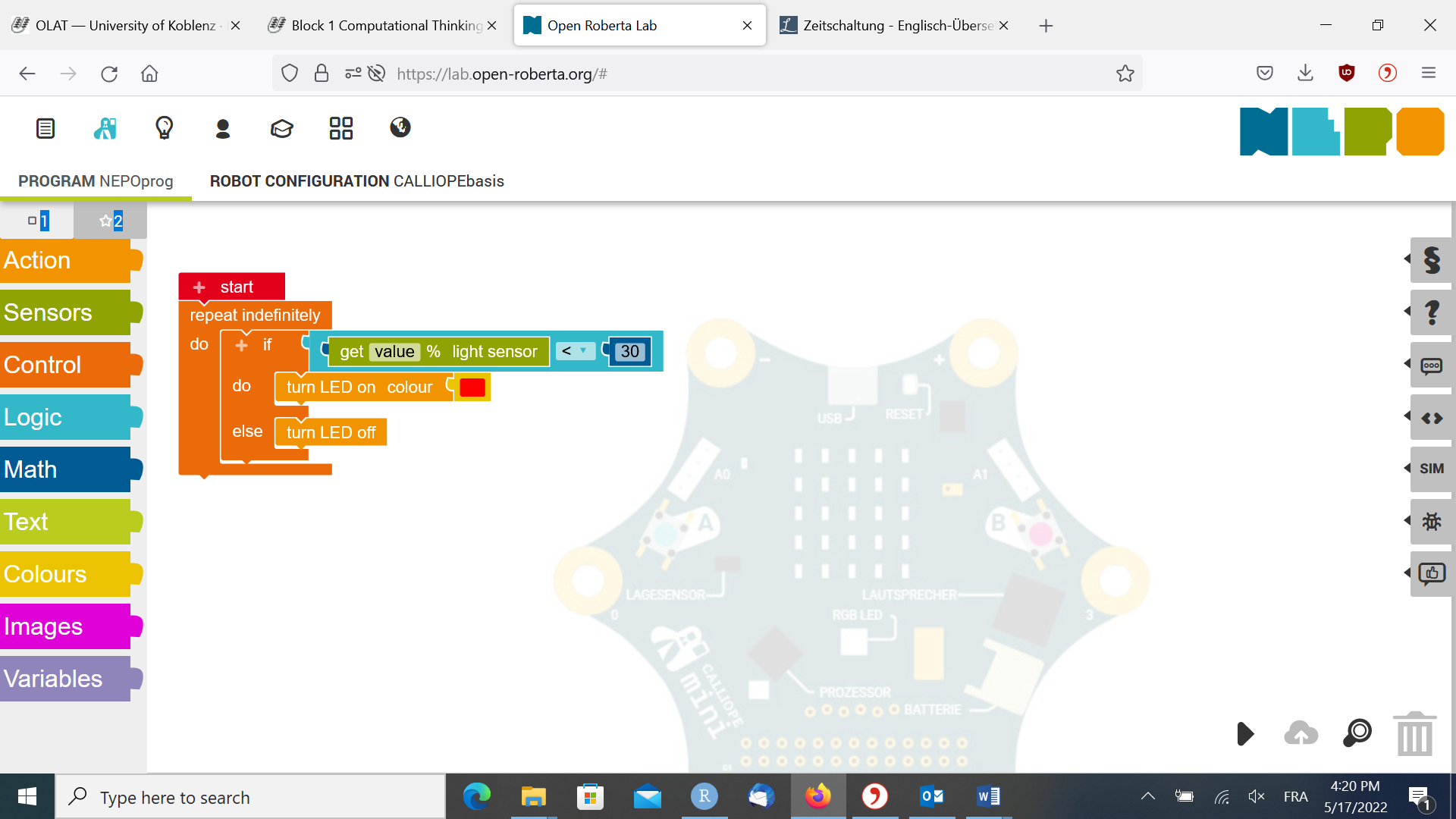


Task 7: Program a dice.


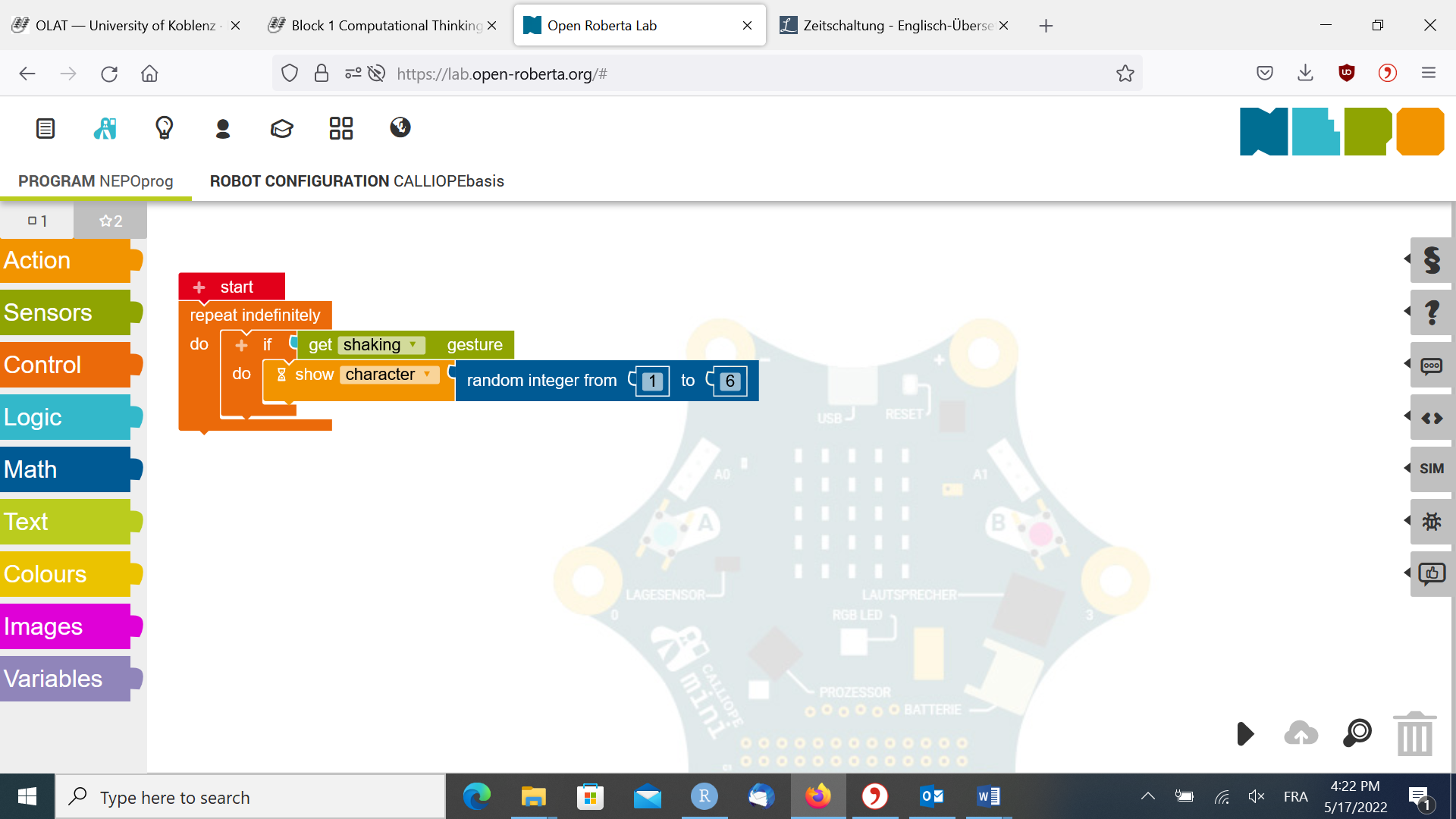


Task 8: Program a temperature warning system that is activated by a sensor and gives a visual and audible alarm when the temperature drops below 4°C.


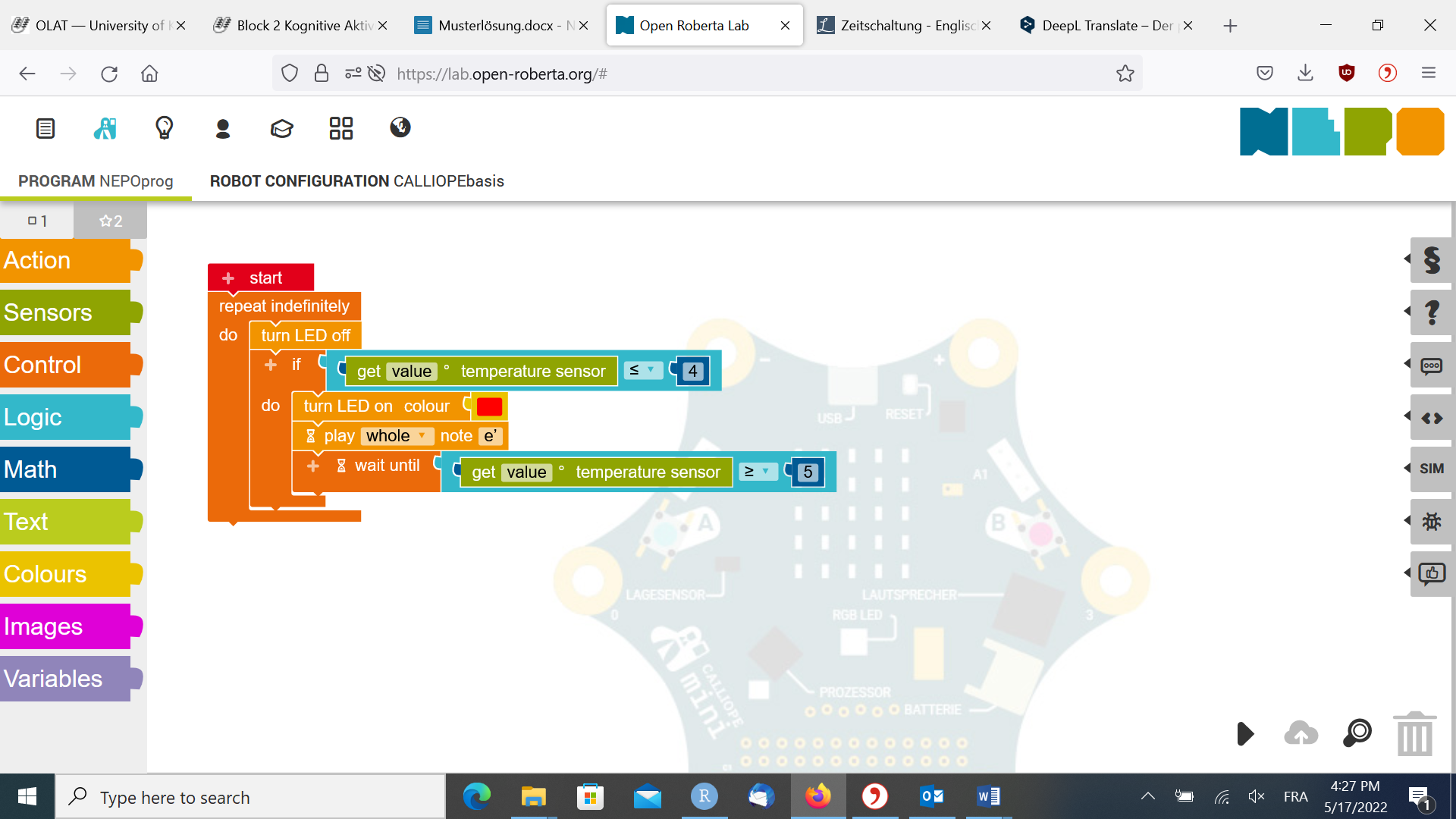


Task 9: Program an alarm system that is activated by a sensor and gives a visual and audible alarm when triggered.


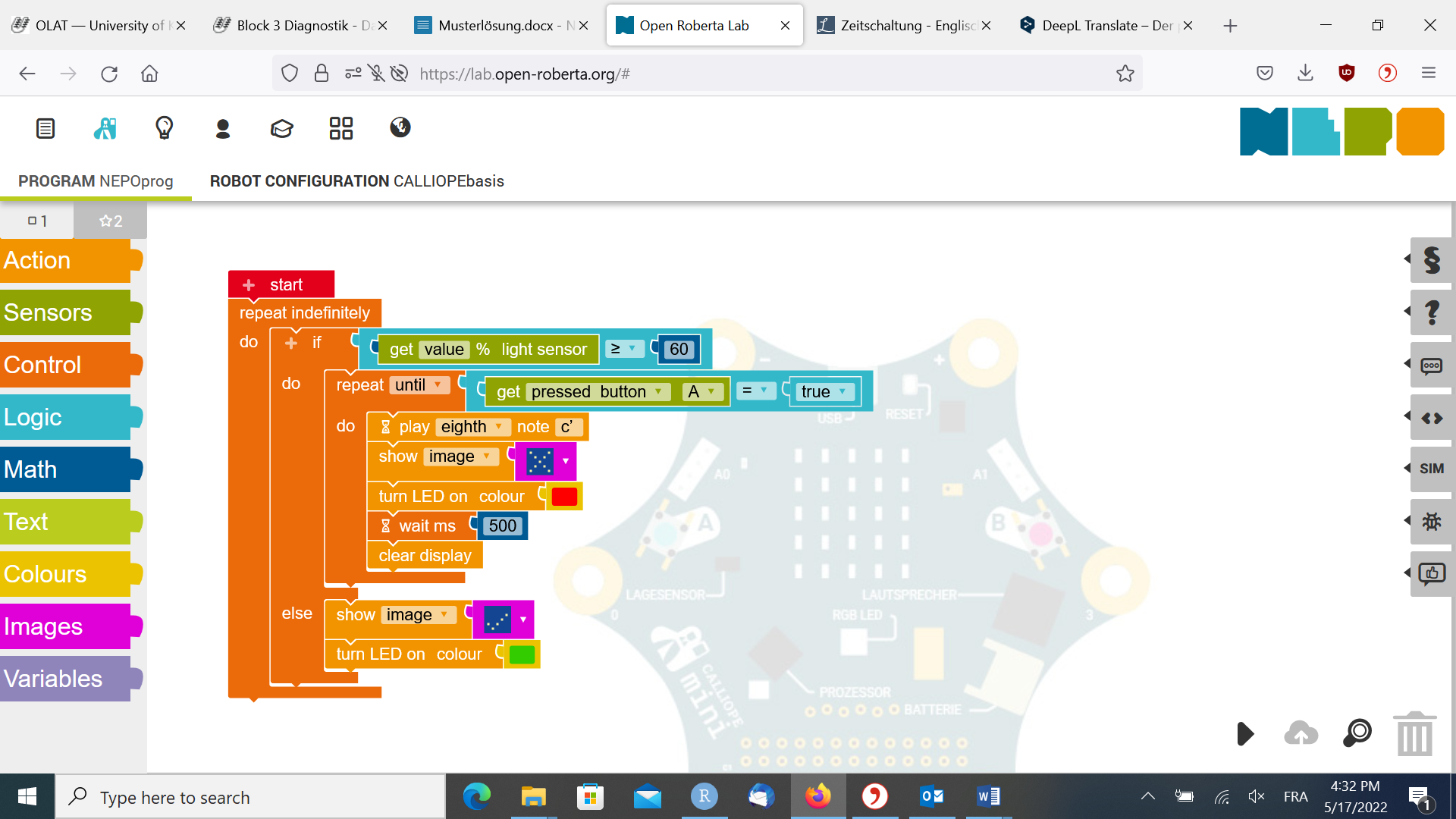


Task 10: Program a metronome.


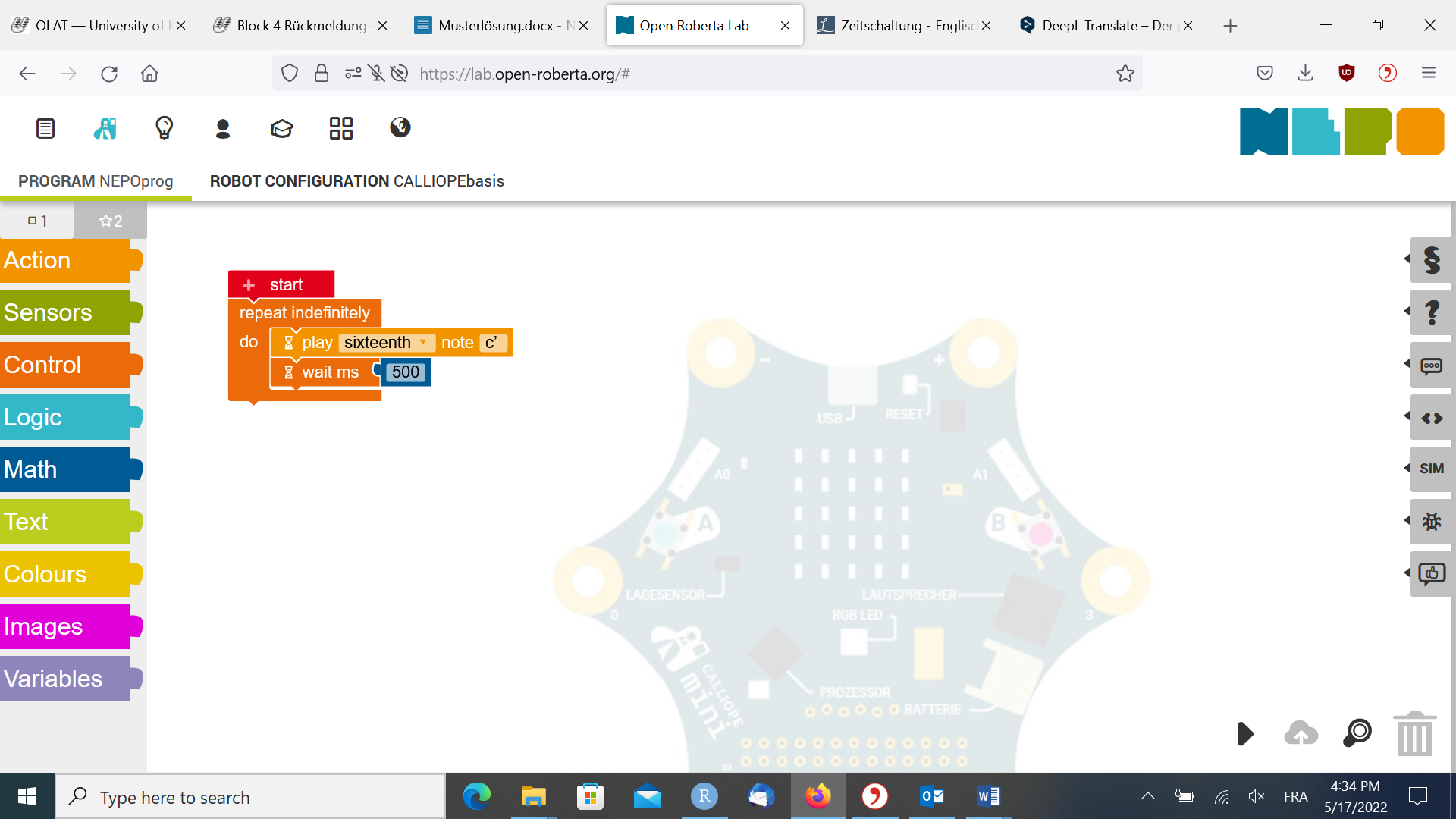


# Supplementary Material 3. Results of the confirmatory factor analyses for the expectancy and value components.

Supplementary Table 2

*Confirmatory factor analyses for the expectancy and value components*

| Scale | Item | λ | CFI | TLI | RMSEA (*p*) |
| --- | --- | --- | --- | --- | --- |
| Self-concept |  |  | .97 | .94 | .06 (.277) |
|  | I am just not good at programming. | .44 |  |  |  |
|  | I understand basics of programming. | .68*** |  |  |  |
|  | I learn programming quickly. | .70*** |  |  |  |
|  | I believe that programming is one of my strengths. | .63*** |  |  |  |
|  | In the seminar, I solve even the most difficult programming tasks (EG).  I solve even the most difficult programming tasks (CG). | .63*** |  |  |  |
| Self-efficacy |  |  | .98 | .95 | .10 (.050) |
|  | I think I can program conditions with Calliope. | .93 |  |  |  |
|  | I think I can program a compass with Calliope. | .94*** |  |  |  |
|  | I think I can transfer my knowledge about programming to another platform (e.g., Bluebot). | .80*** |  |  |  |
|  | I think I can program loops. | .65*** |  |  |  |
| Intrinsic value |  |  | .95 | .89 | .14 (.001) |
|  | I program because I enjoy it. | .45 |  |  |  |
|  | I look forward to programming. | .73*** |  |  |  |
|  | I enjoy programming. | .65*** |  |  |  |
|  | I am interested in the things I learn about programming during the seminar (EG).  I am interested in the things I learn about programming (CG). | .88*** |  |  |  |
|  | I enjoy learning new things about programming. | .89*** |  |  |  |

| Utility Value |  |  | .98 | .94 | .12 (.024) |
| --- | --- | --- | --- | --- | --- |
|  | Making an effort in programming is worth it because it will help me in the work that I will do later on. | .77 |  |  |  |
|  | Learning to program is worthwhile for me because I will teach it to children later. | .85*** |  |  |  |
|  | Programming is important for me because I want to teach it later. | .77*** |  |  |  |
|  | I will learn many things about programming that will help me with my future job. | .80*** |  |  |  |
| Attainment Value |  |  | .95 | .86 | .17 (.000) |
|  | It is important to me that children learn how to write programs. | .80 |  |  |  |
|  | It is important to me that children understand basic algorithms. | .73*** |  |  |  |
|  | It is important to me to teach children the basics of programming. | .79*** |  |  |  |
|  | It is important to me that children understand how a computer “thinks”. | .67*** |  |  |  |
| Emotional Costs |  |  | .94 | .90 | .10 (.003) |
|  | I worry that it will be difficult for me to solve programming tasks. | .73 |  |  |  |
|  | I get very tense when I have to solve programming tasks. | .79*** |  |  |  |
|  | I get nervous when I deal with programming problems. | .82*** |  |  |  |
|  | I feel helpless when doing a programming problem. | .67*** |  |  |  |
|  | I worry that I will not pass the seminar because of bad programming performance (EG).  I worry that I will face disadvantages because of bad programming performance (CG). | .54*** |  |  |  |
|  | I am nervous when I think about that I will have to teach children how to program. | .56*** |  |  |  |

Notes. *p < .05. **p < .01. ***p < .001. All factor loadings are standardized. The first factor loading has no significance level, because it defines the metric of the latent variable (see Eid, Gollwitzer & Schmitt, 2010).
